# Onconase Restores Cytotoxicity in Dabrafenib-Resistant A375 Human Melanoma Cells and Affects Cell Migration, Invasion and Colony Formation Capability

**DOI:** 10.3390/ijms20235980

**Published:** 2019-11-27

**Authors:** Alice Raineri, Sabrina Fasoli, Rachele Campagnari, Giovanni Gotte, Marta Menegazzi

**Affiliations:** Department of Neuroscience, Biomedicine and Movement Sciences, Biological Chemistry Section, University of Verona, Strada Le Grazie 8, I-37134 Verona, Italy; alice.raineri@univr.it (A.R.); sabrina.fasoli@univr.it (S.F.); rachele.campagnari@univr.it (R.C.)

**Keywords:** human melanoma, dabrafenib, chemotherapy resistance, onconase, NF-κB

## Abstract

Melanoma is a lethal tumor because of its severe metastatic potential, and serine/threonine-protein kinase B-raf inhibitors (BRAFi) are used in patients harboring BRAF-mutation. Unfortunately, BRAFi induce resistance. Therefore, we tested the activity of onconase (ONC), a cytotoxic RNase variant, against BRAFi-resistant cells to re-establish the efficacy of the chemotherapy. To do so, an A375 dabrafenib-resistant (A375DR) melanoma cell subpopulation was selected and its behavior compared with that of parental (A375P) cells by crystal violet, 5-Bromo-2’-deoxyuridine incorporation, and cleaved poly(ADP-ribose) polymerase 1 (PARP1) western blot measurements. Then, nuclear p65 Nuclear Factor kappaB (NF-κB) and IκB kinases-α/β (IKK) phosphorylation levels were measured. Gelatin zymography was performed to evaluate metalloproteinase 2 (MMP2) activity. In addition, assays to measure migration, invasion and soft agar colony formation were performed to examine the tumor cell dissemination propensity. ONC affected the total viability and the proliferation rate of both A375P and A375DR cell subpopulations in a dose-dependent manner and also induced apoptotic cell death. Among its pleiotropic effects, ONC reduced nuclear p65 NF-κB amount and IKK phosphorylation level, as well as MMP2 activity in both cell subpopulations. ONC decreased cell colony formation, migration, and invasion capability. Notably, it induced apoptosis and inhibited colony formation and invasiveness more extensively in A375DR than in A375P cells. In conclusion, ONC successfully counteracts melanoma malignancy especially in BRAFi-resistant cells and could become a tool against melanoma recurrence.

## 1. Introduction

Malignant melanoma is a highly aggressive skin cancer that has shown increasing incidence and mortality over recent decades [1]. Melanoma is characterized either by a high degree of plasticity or by severe metastatic potential, and the conventional therapies used in clinics are time-limited and far from being successful [2]. Indeed, although BRAF inhibitors (BRAFi) are the most potent therapeutic drugs approved to counteract the melanoma cases displaying BRAF-mutated genotype [3], their use is not decisive because patients acquire resistance after 8–10 months of treatment [2,4]. In order to investigate the mechanisms involved in the resistance to dabrafenib or vemurafenib BRAFi, several studies have been addressed to select the resistant melanoma cell populations and better characterize their key features [5,6,7,8,9,10]. It has been recently reported that A375 melanoma cells become resistant to vemurafenib upon acquiring a cancer stem cells (CSC) phenotype and, consequently, the ability to form melanospheres [11]. Indeed, melanoma CSC can be identified because they display either undifferentiation or embryonic state markers, such as NGF receptor, Prominin-1 (CD133), Transcription Factor Sox2, Octamer-binding protein 4, and Homeobox protein NANOG (NANOG) [11]. Moreover, CSC are a subpopulation of tumor cells that are extremely malignant, since CSC can infiltrate and colonize other tissues. It was reported that, during vemurafenib treatment, resistant cells rapidly led to tumor onset in mice implanted with a mixture of sensitive and BRAFi-resistant A375 melanoma cells. On the contrary, a tumor does not grow in mice treated with the same drug but implanted with only A375 vemurafenib-sensitive cells [10]. Hence, drug-resistant cells can quickly lose their adherence ability and gain both migratory and invasive capability. This also occurs through the penetration of the cells into the extracellular matrix (ECM), an event that leads to the formation of new tumors in other body sites [12]. Considering that ECM acts as a mechanical barrier against tumor-cell mobility, its degradation becomes crucial in supporting the tumor dissemination process. In this regard, it is well known that matrix metalloproteinases (MMPs) cause ECM damage and that high levels of MMPs are secreted by melanoma cells [13,14]. Therefore, the strategy of blocking melanoma cell migration, invasion, and progression could be aided by drugs inhibiting MMPs activity. Moreover, intracellular signals, such as the one represented by the Wnt/β-catenin pathway, play an important role in melanoma pathogenesis and progression [15,16]. Indeed, Sinnberg et al. demonstrated that β-catenin is involved in the melanoma acquired resistance to vemurafenib [17]. Hence, the nuclear localization of β-catenin could be a specific determinant for the cell resistance to BRAFi.

In order to find new therapeutic strategies against drug resistance, several recent works have focused their investigations on the combination of BRAFi with synthetic compounds or with natural products [14,18,19,20]. In a previous report, we demonstrated that nanomolar concentrations of the enzyme onconase (ONC) exert both cytostatic and cytotoxic activities against A375 melanoma cell line [21]. In addition, ONC is known to counteract the viability of two conjunctival melanoma cell lines [22]. This protein is a secretory, 11.8 kDa basic enzyme extracted from frog oocytes that exerts ribonucleolytic activity and shows the typical fold of the pancreatic-type RNases, as does RNase A [23,24]. Importantly, ONC displays a remarkable antitumor action [24,25] because it is efficiently internalized in the malignant cells. The specificity of ONC for cancer cells depends on its high number of basic charges that eases its binding to the tumor cell surface, which in turn displays a higher amount of negative charges than normal cells [26]. ONC can enter cells by endocytosis in the early endosome and, finally, reaches the cytosol and evades the intracellular RNase inhibitor, thus exerting a cytotoxic action [26]. It is a multi-target drug, since its ribonucleolytic activity against tRNA and miRNA causes multiple biological effects by up- or down-regulating the expression of several genes [26,27]. Notably, ONC has been used as a therapeutic agent in phase II and III clinical trials against non-small cell lung carcinoma and unresectable mesothelioma, respectively [27]. Although being a heterologous protein, low immunogenicity levels have been reported in clinic, as well as minor side effects other than nephrotoxicity that disappears upon halting ONC administration [27]. In malignant mesothelioma, ONC inhibits Tumor Necrosis Factor-alpha (TNFα)-elicited NF-κB nuclear translocation and reduces MMP9 activity, as well as cell invasiveness [28]. In the same neoplasia, the ONC-elicited NF-κB downregulation is caused by changes in the expression levels of two specific miRNAs. In this way, ONC decreases colony formation, motility, invasion, and proliferation of mesothelioma cells [29]. Notably, we have recently shown that this RNase inhibits NF-κB DNA-binding in A375 melanoma cells as well [21].

In the present work, we analyze the ability of ONC to affect the viability of A375 cells which have acquired resistance to dabrafenib (A375DR), in comparison with parental (A375P) cells. Therefore, the impact of ONC on cell proliferation, apoptosis, cell migration, and invasion capability, as well as on the inhibition of NF-κB nuclear translocation and of MMP2 activity, have been compared here within the two A375 cell subpopulations.

## 2. Results

### 2.1. ONC Strongly Affects Cell Viability of both Parental and Dabrafenib Resistant A375 Cells

**Figure 1:**
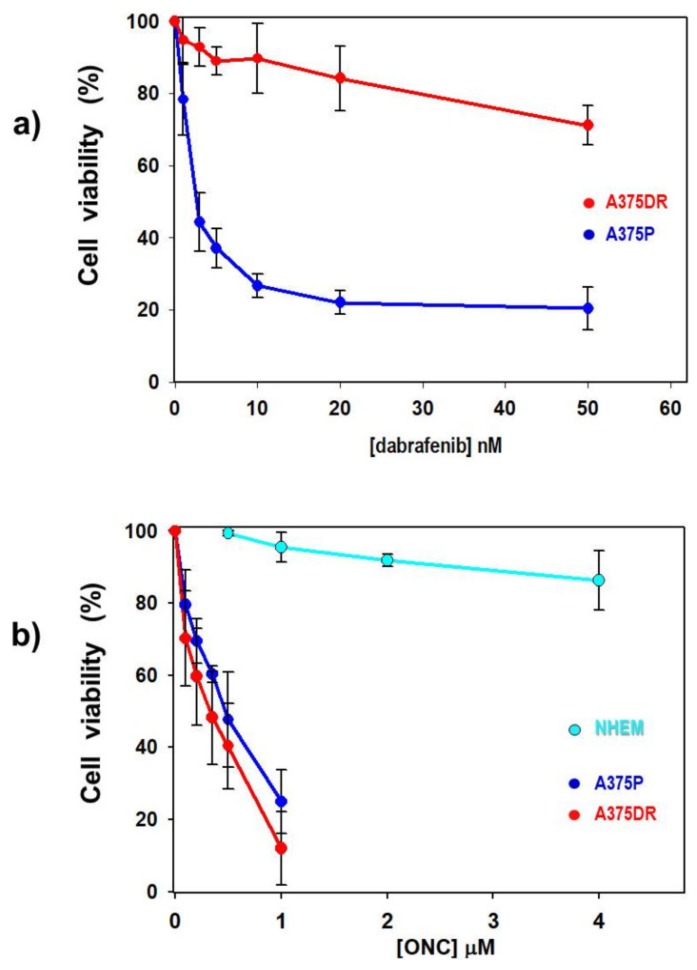
Effect of dabrafenib or onconase (ONC) on the viability of melanoma A375 and of normal human epidermal melanocytes (NHEM) cells. (**a**) A375P (blue dots) and A375DR (red dots) cell viability detected after 72 h incubation with increasing concentrations of dabrafenib. For each dabrafenib concentration tested (panel a), all A375P versus A375DR comparisons are statistically significant (see text). (**b**) cell viability of A375P (blue dots), A375DR (red dots), and NHEM (cyan dots) after 72 h incubation with increasing concentrations of ONC. Statistically significant differences are present (*p* < 0.0001) between NHEM versus A375P or A375DR cells, either at 0.5 or 1 µM ONC, while not between the two A375 cell subpopulations at all ONC concentrations tested. All values reported are the average of four to five independent experiments, each performed in six replicates, ± S.D.

Considering that drug resistance is one of the main factors limiting the efficacy of melanoma therapy, the BRAF-mutated-A375 human melanoma cell line was cultured with gradually increasing concentrations of dabrafenib until a cell subpopulation grew in the constant presence of 5 µM of this drug. Then, the resulting dabrafenib-resistant A375DR cells were maintained for six months under a selective pressure of the drug. After this period, dabrafenib sensitivity was measured by crystal violet assay in both A375P and A375DR cell subpopulations that were treated with dabrafenib concentrations ranging from 1 to 50 nM. As shown in Figure 1a, A375P cells were highly sensitive to dabrafenib, with IC_25_ and IC_50_ of 1.2 and 2.7 nM, respectively. Instead, the A375DR cell viability was not significantly affected up to 20 nM drug concentration, reaching IC_25_ only at 44 nM. Moreover, its relative IC_50_ can be only extrapolated, reaching a value that definitely overpasses 5 µM, i.e., the maximal dabrafenib concentration used in our experiments. Importantly, the differences detected by comparing the data collected from A375P and A375DR cells were statistically significant for each concentration tested (Figure 1a: *p* = 0.02, *p* = 0.0002, *p* = 0.00008, *p* = 0.0001, *p* = 0.0004, and *p* = 0.0001, for 1, 2, 5, 10, 20, and 50 nM dabrafenib, respectively).

In agreement with a recent paper published by our group [21], low ONC concentrations strongly reduced the viability of A375P cells (Figure 1b). In the present work we compare, instead, the effect of ONC registered on parental versus dabrafenib-resistant subpopulations of the same cell line. Figure 1b shows that the viability of both cell subpopulations is reduced to a similar extent, and in a dose-dependent manner, after a 72 h culture with ONC, with calculated IC_50_ values of 0.40 and 0.32 µM for A375P and A375DR cells, respectively. No statistically significant differences in the sensitivity to ONC emerged within the two cell subpopulations, although the mean viability reduction of A375DR cells was lower than that of parental ones for each concentration tested (Figure 1b).

### 2.2. ONC Does not Affect Cell Viability of Normal Melanocytes

To evaluate the specificity of ONC activity against melanoma cells, we also measured the sensitivity of normal human epidermal melanocytes (NHEM) to this RNase variant. NHEM cells were incubated for 72 h with the two ONC concentrations that were the most effective against malignant cells (0.5, 1 µM), and also with 2 and 4 µM ONC (Figure 1b). By the crystal violet assay, we found no reduction in cell viability either at 0.5 or 1 µM ONC concentration (Figure 1b, cyan dots; NHEM versus A375P, *p* = 0.00004 and *p* = 0.00002 for 0.5 and 1 µM ONC, respectively). Moreover, the maximal ONC dose (4 µM), tested exclusively in the NHEM cells, reduced their viability only by 14%. Hence, we conclude that ONC displays quite high cytostatic and cytotoxic effects only in melanoma cells, while not doing so in normal melanocytes.

### 2.3. ONC Decreases the Proliferation Rate of both A375P and A375DR Cell Subpopulations

**Figure 2:**
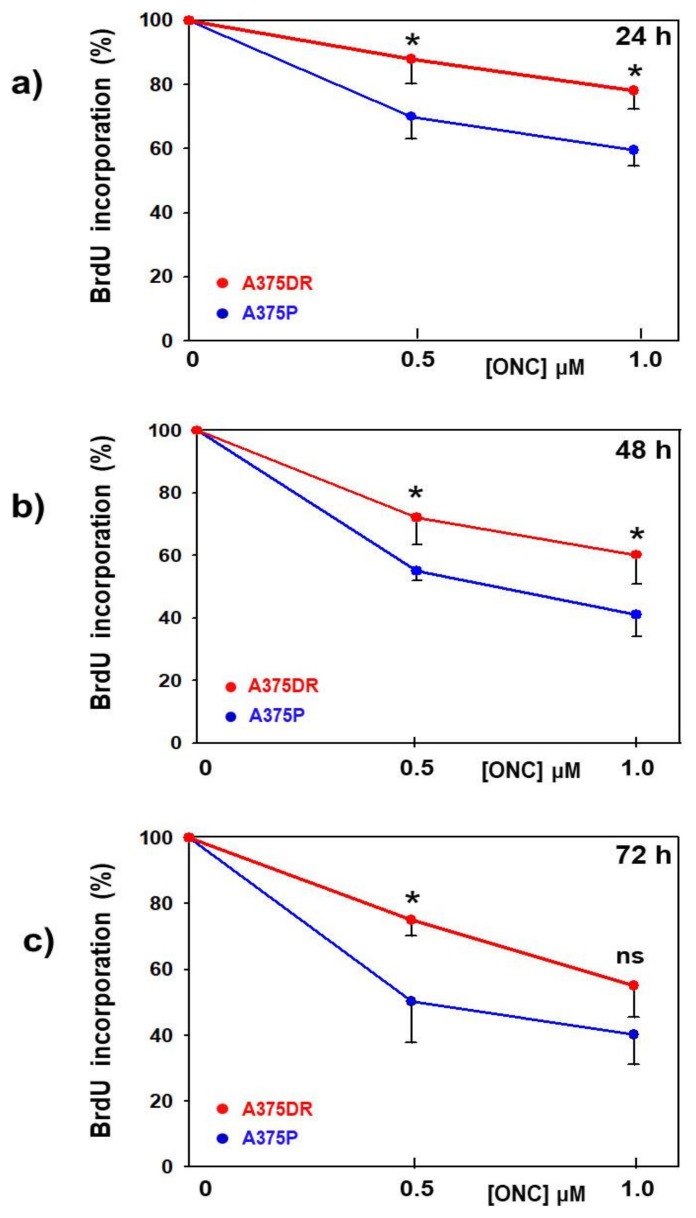
Effect of ONC in the proliferation rate of the two A375 cell subpopulations. (**a**) 24 h, (**b**) 48 h, and (**c**) 72 h measurement of cell proliferation performed with 5-Br-2’-deoxyuridine (BrdU) incorporation in A375P (blue dots) or A375DR (red dots) cells after being incubated with 0.5 or 1.0 µM ONC, in comparison with ONC-free controls. The BrdU data are normalized to each control sample to better visualize the lower reduction of the proliferation rate elicited by ONC in A375DR than in A375P cells. Values reported are the average of four independent experiments, each performed in triplicate, ± S.D. * *p* < 0.05; ns, not statistically significant.

We performed a 5-Br-2’-deoxyuridine (BrdU) incorporation assay to identify whether in both cell subpopulations the viability reduction elicited by ONC might depend on the cell proliferation rate or, instead, on a cell mass decrease consequent to cell death. After 24, 48, and 72 h culture with ONC, an additional 4 h BrdU incubation showed a concentration-dependent reduction of its incorporation in both A375P and A375DR cells. Nevertheless, ONC-treated A375DR cells showed a smaller reduction of BrdU incorporation level than A375P ones, as is clearly visible in Figure 2a–c. In these panels, data have been normalized to each parental or dabrafenib-resistant ONC-free control. All time-point differences emerging by comparing the two ONC-treated cell subpopulations are statistically significant), except for 1 µM ONC at 72 h (A375P versus A375DR for 0.5 and 1 µM ONC, respectively: 24 h, *p* = 0.05, *p* = 0.04; 48 h, *p* = 0.02, *p* = 0.05; and 72 h, *p* = 0.03, n.s.). Although not visible in Figure 2, we underline that also ONC-free A375DR cells appeared to be about 50–70% less proliferating, as a function of time, than ONC-free A375P cells.

In the end, these data indicate that ONC decreases the proliferation rate of both A375P and A375DR cells, although this reduction is more evident in A375P, probably because these cells display a higher replication rate than the A375DR subpopulation.

### 2.4. ONC Induces Apoptotic Cell Death Mainly in A375DR Cells

**Figure 3:**
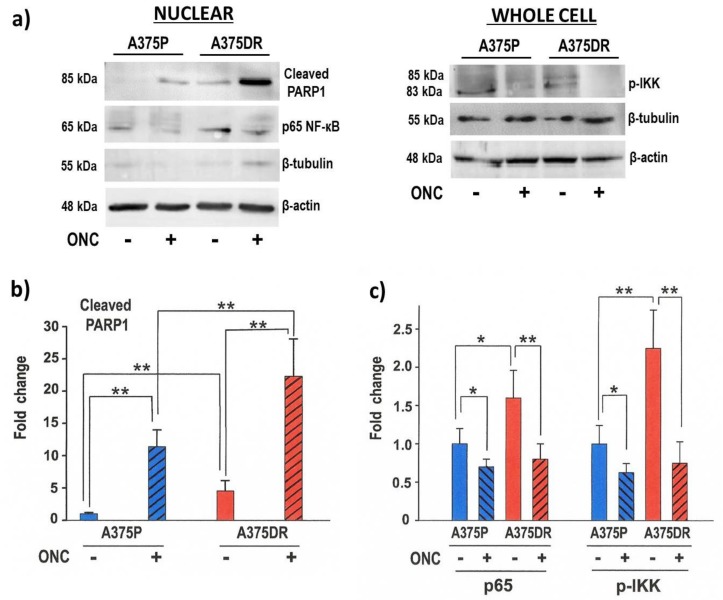
ONC effects in the amount of cleaved PARP1, nuclear p65, and the phosphorylation level of IκB kinases-α/β (p-IKK) in both A375 cell subpopulations. (**a**) Representative WB performed with antibodies recognizing PARP1, p65 in the nucleus and the p-IKK. The hybridization with β-tubulin of either the nuclear or the whole cell protein extractsWB membranes has been performed to exclude the cytosolic contamination in the nuclear extracts. β-actin expression levels were used as the internal control; (**b**) Fold-change for the quantification of the cleaved PARP1 WB bands detected after incubating both cell subpopulations (A375P, blue bars; A375DR, red bars) in the presence (diagonal hatched bars) or absence (no hatching) of ONC; and (**c**) Fold-change for the quantification of p65 nuclear localization and p-IKK WB bands detected after incubating both cell subpopulations in the presence or absence of ONC: Data reported, obtained after β-actin normalization, are the average of four independent WB experiments ± S.D. All differences registered in panels b and c between ONC-free and ONC-treated samples are statistically significant in both cell subpopulations. * *p* < 0.05, ** *p* < 0.01.

Most of the cells treated for 72 h with 1 µM ONC turned out to be detached from the plate, indicating that cell death actually took place. Furthermore, this event was more evident in A375DR than in A375P cells. To verify whether ONC-treated cells underwent apoptosis, the expression level of cleaved PARP1 was measured. In Figure 3a, a representative western blot (WB) is shown, while in Figure 3b the average expression level of cleaved PARP1 relative to four independent experiments, and measured after β-actin normalization, is reported. ONC-free A375DR cells showed higher cleaved PARP1 expression level than ONC-free A375P ones (*p* = 0.01). The amount of cleaved PARP1 increased significantly in all ONC-treated cell samples if compared to each ONC-free one (*p* = 0.002 and *p* = 0.005 for A375P and A375DR, respectively), thus confirming that 1 µM ONC induced the apoptotic death of A375 cells. Remarkably, ONC-treated A375DR cells showed an impressive increase of cleaved PARP1 amount in comparison with ONC-free A375DR cells, but also with ONC-treated A375P (ONC-treated A375DR versus ONC-treated A375P, *p* = 0.01). In conclusion, although ONC-treated A375DR cells showed a lower reduction in the cell proliferation rate than ONC-treated A375P cells (see Figure 2), they appear more prone to undergoing apoptotic cell death than the parental ones (Figure 3b).

### 2.5. ONC Affects NF-κB Activation in both A375 Cell Subpopulations

As measured by WB, ONC-free A375P cells showed detectable amounts of p65 NF-κB localized in the nucleus, and this expression level was even increased in ONC-free A375DR cells (Figure 3a,c, *p* = 0.05), suggesting that NF-κB was activated in both ONC-free subpopulations. Instead, either A375P or A375DR cells treated with 1 µM ONC showed a significantly decreased nuclear p65 level (Figure 3a,c; *p* = 0.04 and *p* = 0.008, for A375P and A375DR, respectively). To exclude the contamination of nuclear extracts by cytosolic proteins, WB membranes containing either the nuclear or the whole cell protein extracts were hybridized with the β-tubulin antibody. As shown in Figure 3a, a large amount of β-tubulin was present in the whole cell protein extracts (right panel), while only very low amounts were visible in the nuclear protein extract samples (left panel). This suggests that the nuclear protein extracts were pure enough to consider the nuclear p65 levels detected reliable. To further confirm this result, we measured the phosphorylation level of IκB kinases-α/β (p-IKK) that triggers NF-κB activation. We found that these kinases follow the same trend shown by p65. Indeed, the detected p-IKK level was higher in ONC-free A375DR than in A375P cells (*p* = 0.008), while ONC decreased its amount especially in A375DR cells (Figure 3a,c; *p* = 0.04 and *p* = 0.001, for A375P and A375DR, respectively). Therefore, our data suggest that ONC reduces p65 NF-κB nuclear localization by inhibiting IκB kinases-α/β (IKK) phosphorylation.

### 2.6. The A375DR Cell Subpopulation Displays the Presence of CSC Biochemical Markers

**Figure 4:**
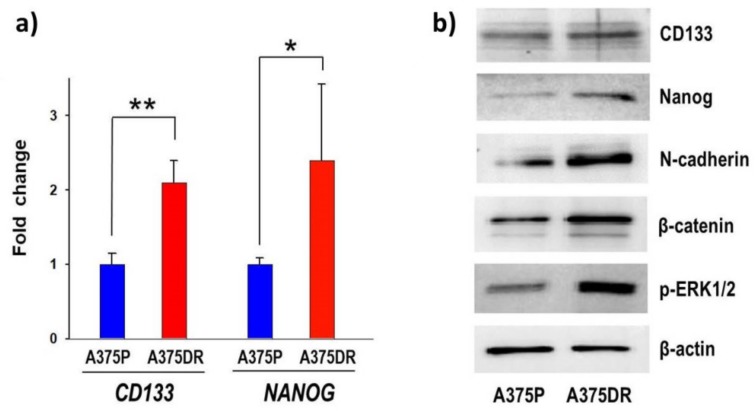
Expression levels of malignancy markers on A375DR versus A375P cells. (**a**) mRNA expression levels of *CD133* and *NANOG*. Values were measured with RT-qPCR in ONC-free A375P (blue bars) and A375DR (red bars) cells, respectively. Average data ± S.D. refer to three independent experiments, each performed in triplicate; * *p* < 0.05, ** *p* < 0.01; and (**b**) representative WB showing the expression levels of CD133, NANOG, N-cadherin, nuclear β-catenin, p-ERK1/2, and β-actin (the latter for normalization) relative to the two ONC-free A375 cell subpopulations.

A375DR cells display a lower BrdU incorporation level than their BRAFi-sensitive A375P counterpart. This behavior may suggest that CSC clonal expansion occurred in this subpopulation [5,30,31]. The mRNA expression levels of *CD133*, a transmembrane glycoprotein expressed on the surface of both normal and cancer stem cells, and of *NANOG*, a transcription regulator factor involved in the maintenance of an embryonic stem cell state, were measured by real-time (RT) qPCR in both A375P and A375DR ONC-free controls. *CD133* and *NANOG* mRNAs showed in A375DR cells a 2.1- and 2.4-fold increases, respectively, in comparison to the relative expression levels detected in A375P, with the amount of the mRNA of TATA box binding protein (*TBP*) used here as the internal standard (Figure 4a; *CD133*, *p* = 0.009 and *NANOG*, *p* = 0.04). These results demonstrate that A375DR cells display a higher gene expression level of both *CD133* and *NANOG* markers of undifferentiation and embryonic stem cells than A375P ones. In addition, the protein amounts of the same CSC markers detected by WB were increased in A375DR cell subpopulation (Figure 4b), although these data need to be further examined in situ by immunofluorescence of flow cytometric analysis.

Thereafter, the protein expression levels of epithelial-to-mesenchymal transition (EMT) markers were measured in both cell subpopulations. Representative WB experiments showing a higher expression level of N-cadherin and nuclear β-catenin in ONC-free A375DR than in ONC-free A375P cell subpopulations are reported in Figure 4b. Our results show that A375DR cells express high levels of CSC mRNA expression markers, such as *CD133* and *NANOG*. This subpopulation undergoes EMT as suggested by the increase of nuclear β-catenin and N-cadherin protein levels. Finally, we confirmed that A375DR cells escape from BRAF inhibition by activating the Extracellular Signal-Regulated Kinase 1 and 2 (ERK1/2) pathway, as shown by the increase of ERK1/2 phosphorylation level (Figure 4b).

### 2.7. ONC Reduces the Ability of both A375P and A375DR Cells to Form Soft Agar Colonies

**Figure 5:**
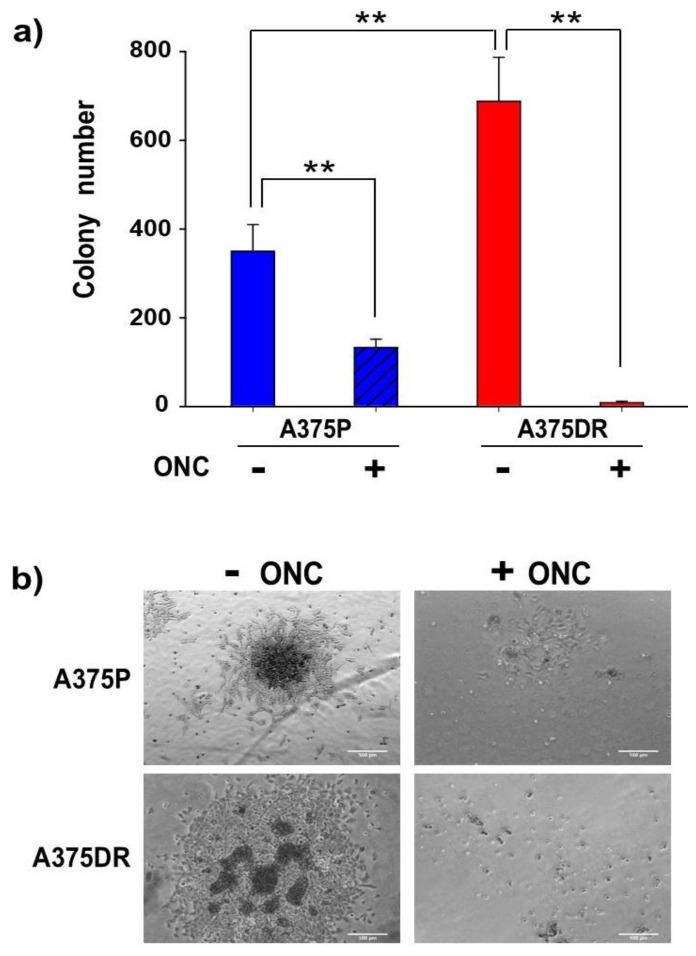
ONC effect on the colony formation of the two A375 cell subpopulations. (**a**) Number of colonies produced in soft agar by A375P (blue bars) and A375DR (red bars) cells after 72 h incubation with or without 1 µM ONC. After ONC treatment, cells were detached, counted, and cultured for 21 days in the medium containing 0.6% agar. Data reported are relative to three independent replicates ± S.D. All differences detected were statistically significant, ** *p* < 0.01; and (**b**) Representative images of the colonies formed by A375P and A375DR cells in the presence or absence of ONC. 10× magnification images were taken with a Zeiss Axio Vert. A1 AxioCAM/cm^−1^ 60N-C1”camera.

Anchorage-independent growth measurements indicate how cells can grow independently of the presence of a solid surface support. Figure 5a shows that, in soft agar, ONC-free A375P cells formed a higher number of colonies than ONC-treated ones (*p* = 0.0008). In addition, the number of ONC-free A375DR colonies formed was very high, while the number of visible colonies approached zero upon 1 µM ONC incubation (*p* = 0.0001). A significant difference was found also between the two ONC-free cell subpopulations (*p* = 0.009). In Figure 5b, representative images of what occurred in soft agar are shown.

A characteristic of stem cells is their capability of forming clones in soft agar [32]. Hence, the remarkable malignancy of A375DR cells is further demonstrated here by their ability to form a high number of colonies in soft agar assay. Importantly, this ability was suppressed by ONC to a greater extent in A375DR than in A375P cells.

### 2.8. ONC Reduces Either Cell Migration Rate or Invasion

**Figure 6:**
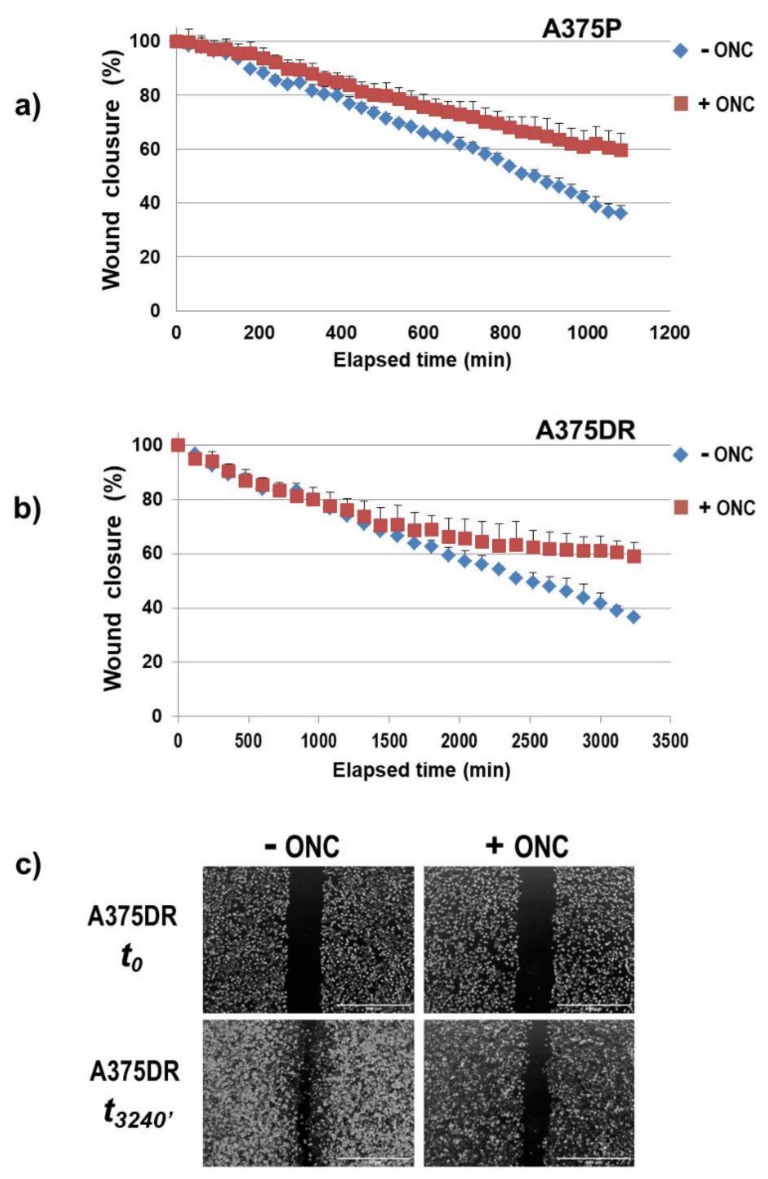
Wound closure time-course of the two A375 cell subpopulations after they suffered scratching. (**a**) A375P and (**b**) A375DR cells; and (**c**) Initial (t_0_) and final (t_3240 min_) frames of ONC-free or 1 µM ONC-treated A375DR cells. 4× magnification images were recorded with the EVOS FL Auto Cell Imaging System.

A wound-healing assay was performed to measure the cell migration ability of both A375P and A375DR subpopulations in the presence or absence of ONC. Notably, the time necessary to close the wound was different: In particular, A375DR cells migrated and proliferated more slowly than the A375P counterpart. Indeed, 1 µM ONC remarkably reduced the migration rate either of A375P or of A375DR cells, as is visible in Figure 6a,b, respectively. The c panel of Figure 6 shows instead the frames recorded either at the beginning or at the end of the test performed with ONC-free or ONC-treated A375DR cells.

**Figure 7:**
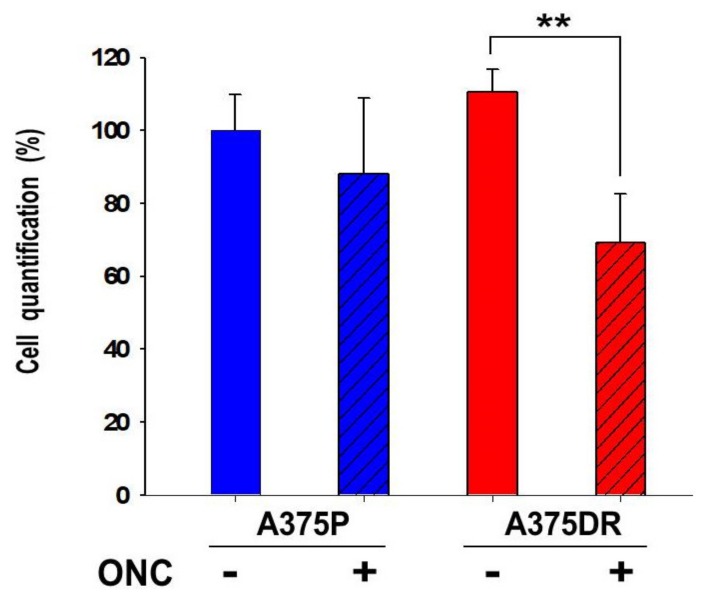
ONC effect on the invasion potential of the two A375 cell subpopulations. A375P (blue bars) and A375DR (red bars) cells treated (diagonal hatched bars), or not (no hatching) with 1 µM ONC for 72 h. Then, cells were detached, counted and seeded in a Transwell plate. After 48 h, they were stained and quantified in the Tecan NanoQuant Infinite M200 Pro Plate reader, as reported in the Materials and Methods Section. ** *p* < 0.01, ONC-treated versus ONC-free A375DR cells. The other differences were not statistically significant.

In order to verify whether A375 cells can acquire an invasive phenotype, we examined the effect of ONC on the ability of A375DR cells to migrate through the Matrigel. Cells were seeded in the upper chamber of a Transwell plate, and complete Dulbecco’s Modified Eagle’s Medium (DMEM) was added to the lower chamber as a chemo-attractant. Then, the invading cells were stained and analyzed after 48 h: Both subpopulations invaded the Matrigel, and ONC-free A375DR cells showed only a small and not significant increase in their invasion potential with respect to the ONC-free A375P subpopulation. Instead, the incubation with 1 µM ONC caused a remarkable 33% reduction in the A375DR cell-invasion capability (*p* = 0.008), while a lower and not statistically significant decrease was found in ONC-treated A375P cells, as shown in Figure 7.

### 2.9. ONC Reduces MMP2 Activity in both A375P and A375DR Cells

**Figure 8:**
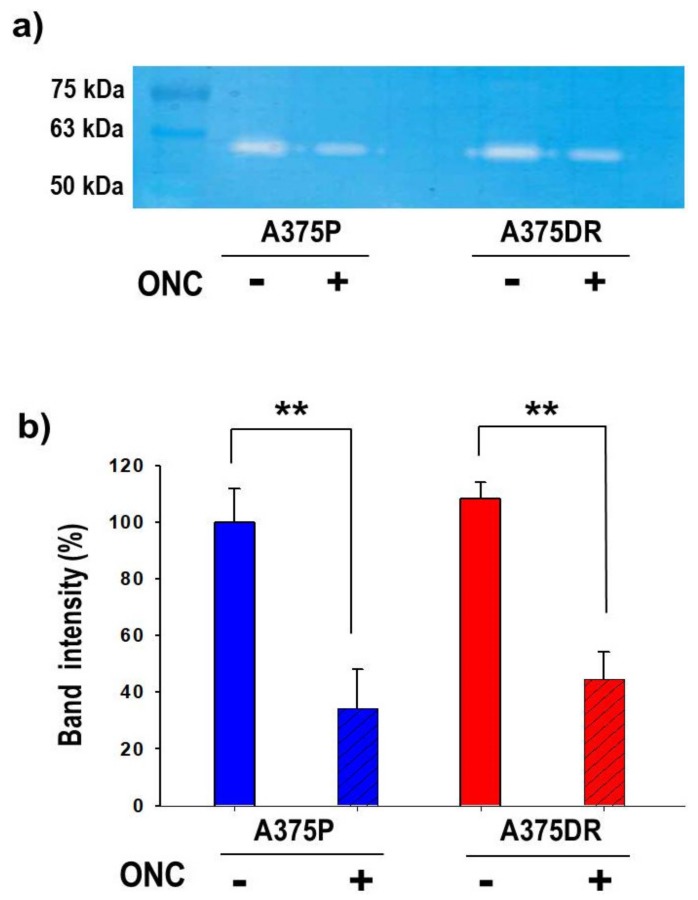
ONC reduced matrix metalloproteinase (MMP)2 activity in both cell subpopulations. (**a**) Representative gelatin-zymography for the evaluation of MMP2 activity; and (**b**) intensity of the digested bands measured with the Image-J software (A375P, blue bars; A375DR, red bars). Values reported are the average of four independent experiments ± S.D. All differences in MMP2 activity found within ONC-treated cells (diagonal hatched bars) and each relative ONC-free control (not hatched) are statistically significant in both cell subpopulations, ** *p* < 0.01.

Both subpopulations were cultured for 48 h with or without 1 µM ONC. Thereafter, cells were serum-starved for 30 h and the conditioned media collected to measure the MMP2 activity by gelatin zymography. The results shown in Figure 8a,b demonstrate that ONC decreases MMP2 activity at a similar and remarkable extent either in A375P or in A375DR cells (*p* = 0.001 and *p* = 0.0006 for A375P and A375DR, respectively).

## 3. Discussion

Targeted therapy against melanoma performed with dabrafenib or vemurafenib BRAFi in patients that harbor BRAF mutations is initially effective but subsequently fails because alternative tumor-growth-inducing pathways are activated. Consequently, resistance to BRAFi is a commonly acquired event in melanoma [33]. In the present study, the aggressive BRAF-mutated A375 human melanoma cell line was used to pave the way for a more effective therapeutic strategy. A subpopulation of A375DR cells was selected after inducing a six-month-long pressure with the constant presence of dabrafenib in the culture medium. The high adaptive/plasticity features of this melanoma cell type favor the selection of more resistant malignant tumor cells expressing stem-like traits that arise when they remain in contact with this drug for a long period. Indeed, A375DR cells showed higher phosphorylated levels of ERK1/2 than A375P ones (Figure 4b), proving that ERK pathway reactivation occurs, in line with other literature data [5,33,34]. In addition, A375DR cells display both higher *CD133* and *NANOG* gene and protein expression levels than A375P ones (Figure 4a). We recall here that an upregulation of the CD133 marker has been recently associated with the capability of forming spheres in culture, a feature that characterizes tumor stem cells [11,31,35]. Indeed, A375DR cells showed here a higher ability to form colonies in soft agar than A375P ones, in confirmation of the data presented by Cordaro et al. [5]. Moreover, the NANOG upregulation detected suggests that A375DR cells acquired pluripotency. This result, along with N-cadherin and β-catenin increases, indicates that mesenchymal features are present in A375DR cells. As reported by Grichnik et al., melanoma CSC display either small size or a low proliferative rate but are still able to initiate a rapidly proliferating cell progeny [36]. Indeed, A375DR cells are less proliferative than A375P, as shown by the BrdU incorporation assay (Figure 2). For this reason, A375DR cells may be less affected by ONC cytostatic activity. In this regard, it had been previously reported that both vemurafenib and encorafenib BRAFi induce senescence in melanoma cells [37,38]. Therefore, it is also possible that a part of the A375DR cell subpopulation did not proliferate since it became senescent and could not respond to the ONC anti-proliferative effect. Importantly, although we found no statistically significant differences in the reduction of the total cell viability within the two ONC-treated A375 subpopulations, the high expression level of cleaved PARP1 found in A375DR cells indicates that ONC elicits a massive apoptotic cell death in this subpopulation. Hence, if a balance between ONC cytostatic and cytotoxic effects seems to take place in parental cells, a switch from an anti-proliferative effect toward the induction of apoptotic cell death is clearly visible in A375DR ones. It is worth noting that a significant difference in the expression of cleaved PARP1 is also detectable between the ONC-free samples of the two cell subpopulations. Therefore, A375DR control cells should be more prone to undergoing apoptotic cell death than A375P, while our results suggest that this unusual propensity is greatly strengthened by ONC.

Indeed, ONC could be more effective in drug association therapy, since it enhances tumor cell sensitivity to several cytotoxic agents displaying different mechanisms of action [21,39,40]. The molecular mechanisms underlying ONC cytotoxicity are linked to its ribonucleolytic activity, since enzymatically inactive variants of this RNase are not cytotoxic [24,41]. Moreover, ONC displays pleiotropic activities because it exerts multi-target actions: In fact, its preferential intracellular targets are tRNAs, although its cytostatic/cytotoxic effects are not ascribable to a generalized protein synthesis inhibition [42]. Furthermore, ONC degrades noncoding RNAs, and, especially, miRNAs [29,39,43]. Indeed, Goparaju et al. demonstrated that ONC modulates the expression of both *hsa-miR-30c* and *hsa-miR-15* that, in turn, cause the inhibition of p50 NF-κB expression and finally reduce pleural mesothelioma malignancy [29]. The propensity of ONC to target miRNAs may justify the large effects exerted on the regulation of gene expression [44]. Again, other papers indicated that ONC inhibits NF-κB activity in both adherent and non-adherent tumor cells [28,45], including A375 ones, as recently reported by our group [21]. In the present work, we found a high p65 NF-κB expression level especially in the nuclear extracts of A375DR cells, but this level was reduced upon ONC administration (Figure 3c). Although the associated molecular mechanisms have not been completely elucidated yet, the ONC effect on the IKK phosphorylation has been clearly detected here. Finally, ONC is known to exert different effects on several intracellular targets [44], and its activity on tumor cells likely depends on the up- or down-regulation balance of many proteins. Therefore, additional proteomics, phospho-proteomics, and miRNASeq approaches could better elucidate these molecular mechanisms of action.

Another aspect to be highlighted is the effect of ONC on A375 tumor cell colony formation, cell migration, and invasion (Figure 5, Figure 6 and Figure 7, respectively). A375DR ONC-free cells formed more colonies in soft agar than ONC-free A375P ones, but the effect of 1 µM ONC is very impressive in the resistant cell subpopulation: Indeed, very few A375DR cells and colonies were present in soft agar upon ONC incubation, suggesting that the effect of ONC was potentiated by a preliminary prolonged treatment with dabrafenib. Instead, the reduction of the colony number detected upon ONC incubation was less remarkable in A375P cells. Again, the cell migration rates of the two A375 subpopulations measured in the wound healing assay were different, indicating that different times are necessary to close the scratch introduced in the cell monolayer. However, the 1 µM ONC incubation reduced the migration of either A375P or A375DR cells, as compared to each relative ONC-free control. As regards the invasion assay, no significant differences were found between the two ONC-free A375P and A375DR counterparts in the ability to cross the Matrigel, in line with data reported by Fratangelo et al. [46]. On the contrary, 1 µM ONC induced a 33% significant reduction of the invasion capability in A375DR cells in comparison with its ONC-free control. Both cell migration and invasion are processes involved in the ability of cells to carry out adhesion and proteolytic degradation of tissue barriers [12,13,14]. Then, the invasion tendency of melanoma cells, and their consequent ability to distribute cellular clones into the metastasis sites are linked to ECM digestion. In addition, the elevated levels of MMPs secreted in ECM by malignant melanoma cells are important determinants in this process. Among several MMPs, MMP2 and MMP9 are crucial because they degrade type IV collagen, the major ECM component [47]. Using RNA-interference, a causal role of MMP2 in invasion had already been demonstrated in human retinal microvascular cells [48], in human extravillous trophoblast [49] and in two hepatocellular carcinoma cell lines [50]. The MMP2 activity measured here in both A375 cell subpopulations with gelatin zymography was strongly reduced by 1 µM ONC (Figure 8). Significantly, Nasu and colleagues demonstrated that ONC is a potent inhibitor of NF-κB activity in malignant mesothelioma, suggesting that the ONC blockage of NF-κB activation leads to the inhibition of MMP9 secretion and of its consequent invasiveness [28]. Again, berberine is also known to inhibit cell viability, as well as migration and invasion of both sensitive and BRAFi-resistant A375 cells, and these effects were associated with a reduction of MMPs activity and with the inhibition of NF-κB and of other signaling pathways [20]. Therefore, all these literature data [20,28] are in line either with the NF-κB nuclear level reduction or with the MMP2 activity inhibition observed in our cell system.

In conclusion, besides its intrinsic cytostatic/cytotoxic effects exerted on parental A375 melanoma cells, ONC significantly enhances its antitumor efficacy by triggering apoptosis in cells that were pre-treated with other drugs. Furthermore, the apoptotic cell death driven by ONC occurs either if a previous treatment did not induce resistance [21] or, as shown in the present work, if the cells had already lost their sensitivity to the cytotoxic effect of a BRAFi. Moreover, being able, in addition to its cytotoxic effect, to counteract both cell migration and invasion by affecting both NF-κB nuclear localization and MMP2 activity, ONC must certainly be regarded as an important tool against tumor metastasis onset.

Last, but not least, we recall that Reck et al. reported a safe and feasible randomized phase III study centered on ONC and doxorubicin (DOX) therapy of unresectable malignant mesothelioma. In this study, however, patients had a significant advantage for survival only when they were previously treated with DOX before they were given ONC [51]. Therefore, the highest sensitivity to ONC of A375DR cells found in our work is in line with this important clinical result [51].

## 4. Materials and Methods

All chemicals, solvents, and buffers were from Sigma-Aldrich (Milan, Italy), unless noted otherwise.

### 4.1. ONC Expression and Purification

The plasmid encoding for recombinant ONC (pcDNA-ONC) was kindly provided by Prof. D. Picone (University of Naples, Federico II, Italy). ONC was expressed in *E. coli*, and extracted and refolded from inclusion bodies. Finally, it was purified following the protocol described by Notomista et al. [52]. Met (-1) was removed by incubating at 37 °C a 0.3 mg/mL ONC solution in 0.2 M potassium phosphate, pH 8.0, containing 20 μM ZnSO_4_, with *P. aeruginosa* aminopeptidase (AAP) at an AAP:ONC = 1:1000 molar ratio. The reaction was blocked after 96 h by adding 0.01 M EDTA, final concentration. Each reaction step was monitored by analyzing the products with SDS-PAGE under reducing conditions. The final chromatographic purification was performed after the removal of the Met (-1) residue with a Superdex 75 HR 10/300 SEC column attached to an ÄKTA-FPLC system (GE-Healthcare, Little Chalfont, UK) [53]. Then, the accuracy of ONC expression was assessed with circular dichroism in a Jasco J-710 spectropolarimeter (Jasco Europe, Cremella (LC), Italy), while the actual Met (-1) removal was detected with Mass Spectrometry (MS) into a Ground Steel MALDI target plate of a Bruker Ultraflextreme MALDI-TOF/TOF instrument (Bruker Daltonics, Billerica, MA, USA) of the “Centro Piattaforme Tecnologiche” (CPT) of the University of Verona, Italy. Finally, with ONC being a pancreatic-type RNase [52,53], its actual enzymatic activity was measured against yeast RNA as a substrate, following the method described by Kunitz [54].

### 4.2. Cell Cultures

Normal human epidermal melanocytes and human melanoma A375 cell lines (ATCC, Manassas, VA, USA), were separately cultured at 37 °C in high glucose DMEM (Gibco, BRL Invitrogen Corp., Carlsbad, CA, USA) supplemented with 10% fetal bovine serum (FBS; Gibco, BRL Invitrogen Corp., Carlsbad, CA, USA), 1% Antibiotic Antimycotic Solution (Gibco, BRL Invitrogen Corp., Carlsbad, CA, USA), and in a 5% CO_2_ humidified atmosphere.

The parental human melanoma A375P cell line was induced to generate dabrafenib resistance (DR) with the aim of selecting a A375DR subpopulation: To do so, A375 cells were cultured at 37 °C with concentrations of dabrafenib (Selleckchem, Aurogene, Rome, Italy) that gradually increased from 1 nM up to 5 µM until a subpopulation grew in the constant presence of this maximal drug concentration. Subsequently, A375DR cells were maintained in culture with 5 µM dabrafenib for six months. Both A375P and A375DR subpopulations were mycoplasma tested (negative) every three months.

### 4.3. Cell Viability Assay

NHEM and both dabrafenib-sensitive parental, A375P, and A375 DR cell subpopulations were seeded in a 96-well plate (2.9 × 10^3^ cells/well). After 24 h culture, A375 cells were incubated with different concentrations of dabrafenib or of ONC, and harvested after 72 h. In a parallel experiment, NHEM cells were treated with ONC to compare their cell viability with the ones of both A375 subpopulations. In the experiments performed with A375DR cells, dabrafenib was removed from the culture medium one day before starting the 72 h incubation with ONC or dabrafenib. To measure cell viability, each cell sample was washed at the end of the treatment with 1× PBS solution (Gibco BRL Invitrogen Corp., Carlsbad, CA, USA), and stained for 5 min with 0.75% crystal violet powder dissolved in 8% formaldehyde and 50% ethanol. Crystal violet was solubilized in PBS/1% SDS solution, and OD_595_ measured in a plate reader (Tecan NanoQuant Infinite M200 Pro, Tecan Group Ltd., Männedorf, Switzerland). Six replicates were performed for each condition/time point.

### 4.4. 5-Br-2’-Deoxyuridine Cell Proliferation Assay

Cell proliferation was assessed with a colorimetric immunoassay based on the measurement of BrdU incorporation during DNA synthesis. A375P or A375DR cells were seeded in 96-well plates (2.9 × 10^3^ cells/well). After 24 h, cells were incubated with or without 0.5 or 1 µM ONC for 24, 48, or 72 h, and, subsequently, for another 4 h with BrdU (BrdU Elisa Kit; Abcam, Milan, Italy). The medium was aspirated and cells were incubated with a fixing solution for 30 min. Plates were washed three times and the anti-BrdU detector antibody was added for 1 h. Plates were then washed a further three times, incubated for 30 min at room temperature with peroxidase goat anti-mouse IgG conjugate and then washed three times again. The tetramethybenzidine chromogenic peroxidase substrate was added, and plates were incubated for 30 min in the dark. Finally, a stop solution was added and the OD_450_ measured in a plate placed in the Tecan NanoQuant Infinite M200 Pro reader (Tecan Group Ltd., Männedorf, Switzerland).

### 4.5. Soft Agar Colony Formation Assay

Cells were seeded in flasks and treated with 1 µM ONC for 72 h. Before seeding, Petri dishes were prepared with soft agar as follows: 1% low gelling temperature agarose and 2× DMEM supplemented with 10% FBS and 1% Antibiotic Antimycotic Solution (Gibco, BRL Invitrogen Corp., Carlsbad, CA, USA) were plated at the bottom layer of the plate, while 0.6% agarose and 2× DMEM supplemented with 10% FBS and 1% Antibiotic Antimycotic Solution were plated on its top. ONC-treated and ONC-free (control) cells were detached, counted and seeded in six-well plates (5000 cells/well), together with the upper soft agar solution. 100 µL/well of fresh medium were added twice a week and cell colonies were stained with crystal violet after 21 days. Semi-solid cultures were performed in triplicate and images were captured with the EVOS FL Auto Cell Imaging System (ThermoFischer Scientific, Waltham, MA, USA).

### 4.6. Wound Closure Cell Migration Assay

A375P cells were seeded in six-well plates (2 × 10^5^ cells/well) and, upon reaching confluence, the monolayer was scratched with a sterile 200 µL pipette tip. A375DR cells were seeded in six-well plates (4 × 10^5^ cells/well) but their monolayer was scratched with a sterile 10 µL pipette tip when they reached confluence. To remove detached cells, wells were washed with complete medium and afterwards refilled with fresh medium containing 10% FBS. 1 µM ONC was added to this culture and samples were compared with the ONC-free ones. A375P cells were incubated for 17 h (1020 min), while A375DR cells for 54 h (3240 min), and the behavior of both cell subpopulations was monitored with EVOS FL Auto Cell Imaging System. Cells were kept at 37 °C in humidified atmosphere with 5% CO_2_ in an EVOS Onstage Incubator (ThermoFisher Scientific, Waltham, MA, USA). The A375P cells movement frames were captured every 30 min over the aforementioned 17 h with a 5× magnification, while the A375DR cells frames every 2 h with a 10× magnification over the aforementioned 54 h, to create time-lapse videos. The relative images were quantitatively analyzed with the Image-J computing software and the MRI Wound Healing Tool (NIH, Bethesda, MD, USA). In the graphics, the mean value relative to each point calculated from four independent experiments is reported ± SD.

### 4.7. Transwell Invasion Assay

Both A375P and A375DR cell subpopulations were incubated for 72 h in the presence or absence of 1 µM ONC. Afterwards, cells were detached with trypsin, counted and seeded in a Transwell composed of polycarbonate membrane inserts of 8 µm pore size, and coated with a uniform layer of a dried basement membrane matrix solution. Cell invasion was measured with the CytoSelect^TM^ cell invasion assay kit (CELL BIOLABS, San Diego, CA, USA), according to the manufacturer instructions. 0.5 mL of complete medium were added as chemoattractant to the lower chamber well, while 0.3 mL of cell suspension consisting of 1 × 10^6^ cells/mL solubilized in DMEM serum-free were seeded on the upper chamber. Invasion capability was analyzed after 48 h: Non-invading cells were removed by cotton swab scrubbing, and the invading cells treated with the staining solution. Finally, the OD_450_ was measured in the Tecan NanoQuant Infinite M200 Pro plate reader (Tecan, Männedorf, Switzerland).

### 4.8. Gelatin Zymography

Both cell subpopulations were cultured in the presence of absence of ONC for 48 h, and then they were serum-starved for 30 h. The conditioned medium was collected and 10× concentrated with an Amicon Ultra-2 Centrifugal Filter Unit (Merck-Millipore, Milan, Italy). Protein concentrations were determined with Coomassie (ThermoFisher Scientific, Waltham, MA, USA) and samples treated with a non-reducing sample buffer. In order to separate proteins, a 7.5% SDS-PAGE was performed. The gel contained 4 mg/mL gelatin and was washed with 50 mM Tris-HCl pH 7.5, 5 mM CaCl_2_, and 1 µM ZnCl_2_ supplemented with 2.5% Triton X-100 (Serva Electrophoresis, Heidelberg, Germany). It was then kept in the incubation buffer (1% Triton X-100, 50 mM Tris-HCl pH 7.5, 5 mM CaCl_2_, and 1 µM ZnCl_2_) for 24 h to allow the digestion by the MMP2 gelatinase. This buffer contained the cofactors necessary to maintain the MMP2 gelatinase activity, so that it could degrade the gelatin present in the gel. The gel was stained with 0.5% Coomassie Brilliant Blue R-250 in 20% ethanol/10% acetic acid, and the gelatinase activity was detected by evaluating presence and intensity of clear bands on the blue background.

### 4.9. Total and Nuclear Protein Purification

In order to purify the total protein content, cell pellets were re-suspended in ~50–100 μL of RIPA lysis buffer (150 mM NaCl, 5 mM EDTA pH 8.0, 50 mM Tris pH 8.0, 1% NP-40, 0.5% sodium deoxycholate, and 0.1% SDS), in the presence of protease and phosphatase inhibitors (Proteoloc protease inhibitor cocktails, and phosphatase inhibitor cocktail 2, respectively; Tema Ricerca, Bologna, Italy). Samples were incubated on ice for 20 min and centrifuged at 25,000× g for 25 min, at 4 °C. The supernatant, containing soluble proteins, was collected and maintained at −80 °C until use. Nuclear extracts were prepared as described in [55] in the presence of protease and phosphatase inhibitors.

OD relative to protein concentrations were measured at 595 nm by the Coomassie brillant blue G-250 Bradford assay in a Jasco V-650 spectrophotometer (Jasco Europe, Cremella (LC), Italy). In order to prepare samples for WB analysis, a quarter of the reducing buffer volume (sample buffer: 625 mM Tris-HCl pH 6.8, 2% SDS, 8% glycerol, 0.25% bromophenol blue, and 5% β-mercaptoethanol) and water were added to 40 μg of total protein extract or to 15 μg of nuclear protein extract. Samples were then boiled for 3 min and placed on ice to aid in protein denaturation.

### 4.10. Quantitative Real Time-PCR Analysis

RNA was extracted with the Pure link RNA kit (Ambion, Invitrogen Corp., Carlsbad, CA, USA), quantified with the Nanodrop UV-vis spectrophotometer (ThermoFisher Scientific, Milan, Italy), and its quality checked by agarose gel electrophoresis. Reverse transcription was performed (500 ng RNA in 10 µL) by using the SuperScript VILO cDNA synthesis kit (Invitrogen Corp., Carlsbad, CA, USA), according to the manufacturer instructions. The expression levels of specific genes were determined with RT-qPCR by using the SensiFAST Sybr No-ROX kit (BIOLINE, London, UK). In each sample, the gene expression level was normalized to the one of the TATA-box binding protein mRNA. PCR reactions were performed in triplicate in a Rotorgene Q (Qiagen, Milan, Italy), with the following amplification primers:

*CD133*:(Fw) 5’GCATTGGCATCTTCTATGGTT3’, (Rev) 5’CGCCTTGTCCTTGGTAGTGT-3’*NANOG*:(Fw) 5’AGTCCCAAAGGCAAACAACCCAGTTC3’, (Rev) 5’TGCTGGAGGCTGAGGTATTTCTGTCTC-3’*TBP*:(Fw) 5’TGTATCCACAGTGAATCTTGG-3’, (Rev) 5’-ATGATTACCGCAGCAAACC-3’

A comparative quantification of gene expression levels was performed with a Pfaffl’s efficiency corrected calculation [56].

### 4.11. WB Assays

Protein extracts were electrophoresed by a 5–10% polyacrylamide SDS-PAGE, and then transferred to a polyvinylidene difluoride membrane (PVDF; Merck-Millipore, Milan, Italy). Membranes were blocked at RT with TBST buffer (10 mM Tris-HCl pH 7.5, 100 mM NaCl, and 0.1% Tween 20 containing 5% bovine albumin serum (BSA, Serva Electrophoresis GmbH, Heidelberg, Germany) for 1 h. Then, they were incubated overnight on a shaker, at 4 °C, with a 5% BSA solution containing the primary antibodies for: Cleaved PARP1 (#5625, 1:2000), p65 NF-κB (#8242S, 1:1000), p-IKKα/β (Ser176/180) (#2697S, 1:1000), pERK1/2 (#4370S, 1:1000) (Cell Signaling Technology, Danvers, CO, USA), β-catenin (GTX101435, 1:5000), N-cadherin (GTX127345, 1:3000), NANOG (GTX100863, 1:3000), CD133 (GTX100567, 1:5000) (Genetex, San Antonio, TX, USA), and β-tubulin (T8328, 1:2000; Sigma-Aldrich, Milan, Italy). Then, membranes were washed three times with TBST buffer for 30 min, then incubated for 1 h with horseradish peroxidase-conjugated secondary antibody (anti-rabbit 1:4000, Cell Signaling Technology, Danvers, CO, USA), and washed with TBST three times again. All protein extracts were normalized with β-actin protein antibody (#4970, 1:1000; Cell Signaling Technology, Danvers, CO, USA). Immuno-detection was carried out with the ECL kit (GE-Healthcare, Little Chalfont, UK) and chemiluminescence signals were detected with ChemiDoc (Bio-Rad, Hercules, CA, USA).

### 4.12. Statistics

All the results are reported as a mean value ± SD. Unless noted otherwise, *p* values were determined using unpaired, two-tailed Student’s t test. For each type of experiment, a minimum of three independent biological replicates were performed.

## References

[1] Franklin C., Livingstone E., Roesch A., Schilling B., Schadendorf D. (2017). Immunotherapy in melanoma: Recent advances and future directions. Eur. J. Surg. Oncol..

[2] Luebker S.A., Koepsell S.A. (2019). Diverse Mechanisms of BRAF Inhibitor Resistance in Melanoma Identified in Clinical and Preclinical Studies. Front. Oncol..

[3] Mattia G., Puglisi R., Ascione B., Malorni W., Carè A., Matarrese P. (2018). Cell death-based treatments of melanoma: Conventional treatments and new therapeutic strategies. Cell Death Dis..

[4] Das Thakur M., Salangsang F., Landman A.S., Pryer N.K., Levesque M.P., Dummer R., McMahon M., Stuart D.D. (2013). Modelling vemurafenib resistance in melanoma reveals a strategy to forestall drug resistance. Nature.

[5] Cordaro F.G., De Presbiteris A.L., Camerlingo R., Mozzillo N., Pirozzi G., Cavalcanti E., Manca A., Palmieri G., Cossu A., Ciliberto G. (2017). Phenotype characterization of human melanoma cells resistant to dabrafenib. Oncol. Rep..

[6] Caporali S., Alvino E., Lacal P.M., Levati L., Giurato G., Memoli D., Caprini E., Antonini Cappellini G.C., D’Atri S. (2016). Targeting the PI3K/AKT/mTOR pathway overcomes the stimulating effect of dabrafenib on the invasive behavior of melanoma cells with acquired resistance to the BRAF inhibitor. Int. J. Oncol..

[7] Caporali S., Alvino E., Lacal P.M., Ruffini F., Levati L., Bonmassar L., Scoppola A., Marchetti P., Mastroeni S., Antonini Cappellini G.C. (2017). Targeting the *PTTG1* oncogene impairs proliferation and invasiveness of melanoma cells sensitive or with acquired resistance to the BRAF inhibitor dabrafenib. Oncotarget.

[8] Fofaria N.M., Frederick D.T., Sullivan R.J., Flaherty K.T., Srivastava S.K. (2015). Overexpression of Mcl-1 confers resistance to BRAF^V600E^ inhibitors alone and in combination with MEK1/2 inhibitors in melanoma. Oncotarget.

[9] Szász I., Koroknai V., Kiss T., Vízkeleti L., Ádány R., Balázs M. (2019). Molecular alterations associated with acquired resistance to BRAF^V600E^ targeted therapy in melanoma cells. Melanoma Res..

[10] Obenauf A.C., Zou Y., Ji A.L., Vanharanta S., Shu W., Shi H., Kong X., Bosenberg M.C., Wiesner T., Rosen N. (2015). Therapy-induced tumour secretomes promote resistance and tumour progression. Nature.

[11] Marzagalli M., Raimondi M., Fontana F., Montagnani Marelli M., Moretti R.M., Limonta P. (2019). Cellular and molecular biology of cancer stem cells in melanoma: Possible therapeutic implications. Semin. Cancer Biol..

[12] Yilmaz M., Christofori G., Lehembre F. (2007). Distinct mechanisms of tumor invasion and metastasis. Trends Mol. Med..

[13] Nikkola J., Vihinen P., Vuoristo M.S., Kellokumpu-Lehtinen P., Kahari V.M., Pyrhonen S. (2005). High serum levels of matrix metalloproteinase-9 and matrix metalloproteinase-1 are associated with rapid progression in patients with metastatic melanoma. Clin. Cancer Res..

[14] Malaponte G., Zacchia A., Bevelacqua Y., Marconi A., Perrotta R., Mazzarino M.C., Cardile V., Stivala F. (2010). Co-regulated expression of matrix metalloproteinase-2 and transforming growth factor-beta in melanoma development and progression. Oncol. Rep..

[15] Xuea G., Romano E., Massic D., Mandalà M. (2016). Wnt/b-catenin signaling in melanoma: Preclinical rationale and novel therapeutic insights. Cancer Treat. Rev..

[16] Lin Y., Wang F., Xing Q., Guo F., Wang M., Li Y. (2018). The biological effect and mechanism of the Wnt/β-catenin signaling pathway on malignant melanoma A375 cells. Exp. Ther. Med..

[17] Sinnberg T., Makino E., Krueger M.A., Velic A., Macek B., Rothbauer U., Groll N., Pötz O., Czemmel S., Niessner H. (2016). Nexus Consisting of Beta-Catenin and Stat3 Attenuates BRAF Inhibitor Efficacy and Mediates Acquired Resistance to Vemurafenib. EBioMedicine.

[18] Greger J.G., Eastman S.D., Zhang V., Bleam M.R., Hughes A.M., Smitheman K.N., Dickerson S.H., Laquerre S.G., Liu L., Gilmer T.M. (2012). Combinations of BRAF, MEK, and PI3K/mTOR inhibitors overcome acquired resistance to the BRAF inhibitor GSK2118436 dabrafenib, mediated by NRAS or MEK mutations. Mol. Cancer Ther..

[19] Hong S.K., Starenki D., Wu P.K., Park J.I. (2017). Suppression of B-Raf^V600E^ melanoma cell survival by targeting mitochondria using triphenyl-phosphonium-conjugated nitroxide or ubiquinone. Cancer Biol. Ther..

[20] Liu J.F., Lai K.C., Peng S.F., Maraming P., Huang Y.P., Huang A.C., Chueh F.S., Huang W.W., Chung J.G. (2018). Berberine Inhibits Human Melanoma A375.S2 Cell Migration and Invasion via Affecting the FAK, uPA, and NF-κB Signaling Pathways and Inhibits PLX4032 Resistant A375.S2 Cell Migration In Vitro. Molecules.

[21] Raineri M., Prodomini S., Fasoli S., Gotte G., Menegazzi M. (2019). Influence of onconase in the therapeutic potential of PARP inhibitors in A375 malignant melanoma cells. Biochem. Pharmacol..

[22] Westekemper H., Freistuehler M., Bornfeld N., Steuhl K.P., Scheulen M., Hilger R.A. (2019). Chemosensitivity of conjunctival melanoma cell lines to target-specific chemotherapeutic agents. Graefes Arch. Clin. Exp. Ophthalmol..

[23] Ardelt W., Mikulski S.M., Shogen K. (1991). Amino acid sequence of an anti-tumor protein from Rana pipiens oocytes and early embryos. Homology to pancreatic ribonucleases. J. Biol. Chem..

[24] Ardelt W., Shogen K., Darzynkiewicz Z. (2008). Onconase and amphinase, the antitumor ribonucleases from Rana pipiens oocytes. Curr. Pharm. Biotechnol..

[25] Ardelt W., Ardelt B., Darzynkiewicz Z. (2009). Ribonucleases as potential modalities in anticancer therapy. Eur. J. Pharmcol..

[26] Rutkoski T.J., Raines R.T. (2008). Evasion of ribonuclease inhibitor as a determinant of ribonuclease cytotoxicity. Curr. Pharm. Biotechnol..

[27] Costanzi D., Sidransky A., Navon H., Goldsweig H. (2005). Ribonucleases as a novel pro-apoptotic anticancer strategy: Review of the preclinical and clinical data for ranpirnase. Cancer Investig..

[28] Nasu M., Carbone M., Gaudino G., Ly B.H., Bertino P., Shimizu D., Morris P., Pass H.I., Yang H. (2011). Ranpirnase Interferes with NF-κB Pathway and MMP9 Activity, Inhibiting Malignant Mesothelioma Cell Invasiveness and Xenograft Growth. Genes Cancer..

[29] Goparaju C.M., Blasberg J.D., Volinia S., Palatini J., Ivanov S., Donington J.S., Croce C., Carbone M., Yang H., Pass H.I. (2011). Onconase mediated NFKβ downregulation in malignant pleural mesothelioma. Oncogene.

[30] Roesch A., Fukunaga-Kalabis M., Schmidt E.C., Zabierowski S.E., Brafford P.A., Vultur A., Basu D., Gimotty P., Vogt T., Herlyn M. (2010). A temporarily distinct subpopulation of slow-cycling melanoma cells is required for continuous tumor growth. Cell.

[31] Roesch A. (2015). Melanoma stem cells. J. Dtsch. Dermatol. Ges..

[32] Dou J., Pan M., Wen P., Li Y., Tang Q., Chu L., Zhao F., Jiang C., Hu W., Hu K. (2007). Isolation and Identification of Cancer Stem-Like Cells from Murine Melanoma Cell Lines. Cell. Mol. Immunol..

[33] Wagle N., Emery C., Berger M.F., Davis M.J., Sawyer A., Pochanard P., Kehoe S.M., Johannessen C.M., Macconaill L.E., Hahn W.C. (2011). Dissecting therapeutic resistance to RAF inhibition in melanoma by tumor genomic profiling. J. Clin. Oncol..

[34] Cvetanova B., Shen Y.C., Shyur L.F. (2019). Cumingianoside A, a phyto-triterpenoid saponin inhibits acquired BRAF inhibitor resistant melanoma growth via programmed cell death. Front. Pharmcol..

[35] Calvani M., Bianchini F., Taddei M.L., Becatti M., Giannoni E., Chiarugi P., Calorini L. (2016). Etoposide-Bevacizumab a new strategy against human melanoma cells expressing stem-like traits. Oncotarget.

[36] Grichnik J.M., Burch J.A., Schulteis R.D., Shan S., Liu J., Darrow T.L., Vervaert C.E., Seigler H.F. (2006). Melanoma, a tumor based on a mutant stem cell?. J. Investig. Dermatol..

[37] Haferkamp S., Borst A., Adam C., Becker T.M., Motschenbacher S., Windhövel S., Hufnagel A.L., Houben R., Meierjohann S. (2013). Vemurafenib induces senescence features in melanoma cells. J. Investig. Dermatol..

[38] Li Z., Jiang K., Zhu X., Lin G., Song F., Zhao Y., Piao Y., Liu J., Cheng W., Bi X. (2016). Encorafenib (LGX818), a potent BRAF inhibitor, induces senescence accompanied by autophagy in BRAF^V600E^ melanoma cells. Cancer Lett..

[39] Zhao H., Ardelt B., Ardelt W., Shogen K., Darzynkiewicz Z. (2008). The cytotoxic ribonuclease onconase targets RNA interference (siRNA). Cell Cycle.

[40] Rybak S.M., Pearson J.W., Fogler W.E., Volker K., Spence S.E., Newton D.L., Mikulski S.M., Ardelt W., Riggs C.W., Kung H.F. (1996). Enhancement of vincristine cytotoxicity in drug-resistant cells by simultaneous treatment with onconase, an antitumor ribonuclease. J. Natl. Cancer Inst..

[41] Boix E., Wu Y., Vasandani V.M., Saxena S.K., Ardelt W., Ladner J., Youle R.J. (1996). Role of the N terminus in RNase A homologues: Differences in catalytic activity, ribonuclease inhibitor interaction and cytotoxicity. J. Mol. Biol..

[42] Iordanov M.S., Ryabinina O.P., Wong J., Dinh T.H., Newton D.L., Rybak S.M., Magun B.E. (2000). Molecular determinants of apoptosis induced by the cytotoxic ribonuclease onconase: Evidence for cytotoxic mechanisms different from inhibition of protein synthesis. Cancer Res..

[43] Qiao M., Zu L.D., He X.H., Shen R.L., Wang Q.C., Liu M.F. (2012). Onconase downregulates microRNA expression through targeting microRNA precursors. Cell Res..

[44] Altomare D.A., Rybak S.M., Pei J., Maizel J.V., Cheung M., Testa J.R., Shogen K. (2010). Onconase responsive genes in human mesothelioma cells: Implications for an RNA damaging therapeutic agent. BMC Cancer.

[45] Tsai S.Y., Ardelt B., Hsieh T.C., Darzynkiewicz Z., Shogen K., Wu J.M. (2004). Treatment of Jurkat acute T-lymphocytic leukemia cells by onconase (Ranpirnase) is accompanied by an altered nucleocytoplasmic distribution and reduced expression of transcription factor NF-kappaB. Int. J. Oncol..

[46] Fratangelo F., Camerlingo R., Carriero M.V., Pirozzi G., Palmieri G., Gentilcore G., Ragone C., Minopoli M., Ascierto P.A., Motti M.L. (2018). Effect of ABT888 on the apoptosis, motility and invasiveness of BRAFi-resistant melanoma cells. Int. J. Oncol..

[47] Moro N., Mauch C., Zigrino P. (2014). Metalloproteinases in melanoma. Eur. J. Cell. Biol..

[48] Gu Y., Ke G., Wang L., Gu Q., Zhou E., He Q., Wang S. (2015). Silencing Matrix Metalloproteinases 9 and 2 inhibits human retinal microvascular endothelial cell invasion and migration. Ophthalmic Res..

[49] Peng B., Zhu H., Klausen C., Ma L., Wang Y., Leung P.C.K. (2016). GnRH regulates trophoblast invasion via RUNX2-mediated MMP2/9 expression. Mol. Hum. Reprod..

[50] Liu J., Li X., Huang J., Liu Y. (2019). Matrix metalloproteinase 2 knockdown suppresses the proliferation of HepG2 and Huh7 cells and enhances cisplatin effect. Open Med..

[51] Reck M., Krzakoski M., Jassem J., Eschbach C., Kozielski J., Costanzi J.J., Gatzemeier U., Shogen K., von Pawel J. (2009). Randomized, multicenter phase III study of ranpirnase plus doxorubicin (DOX) versus DOX in patients with unresectable malignant mesothelioma (MM). J. Clin. Oncol..

[52] Notomista E., Cafaro V., Fusiello R., Bracale A., D’Alessio G., Di Donato A. (1999). Effective expression and purification of recombinant onconase, an antitumor protein. FEBS Lett..

[53] Fagagnini A., Pica A., Fasoli S., Montioli R., Donadelli M., Cordani M., Butturini E., Acquasaliente L., Picone D., Gotte G. (2017). Onconase dimerization through 3D domain swapping: Structural investigations and increase in the apoptotic effect in cancer cells. Biochem. J..

[54] Kunitz M. (1946). A spectrophotometric method for the measurement of ribonuclease activity. J. Biol. Chem..

[55] Gregorelli A., Sgarbossa A., Khan S., Soriente A., De Rosa M., Saturnino C., Menegazzi M. (2016). Tree arachidonoylamide derivatives inhibit pro-inflammatory genes expression by modulating NF-kB and AP1 activity. Med. Chem..

[56] Pfaffl M.W., Horgan G.W., Dempfle L. (2002). Relative expression software tool (REST) for group-wise comparison and statistical analysis of relative expression results in real-time PCR. Nucleic Acids Res..

